# The Study of Visual-Auditory Interactions on Lower Limb Motor Imagery

**DOI:** 10.3389/fnins.2018.00509

**Published:** 2018-07-24

**Authors:** Zhongliang Yu, Lili Li, Jinchun Song, Hangyuan Lv

**Affiliations:** ^1^School of Mechanical Engineering and Automation, Northeastern University, Shenyang, China; ^2^School of Physics, Liaoning University, Shenyang, China

**Keywords:** visual-auditory interactions, motor imagery, mirror neurons, brain-computer interface, event-related desynchronization

## Abstract

In order to improve the activation of the mirror neuron system and the ability of the visual-cued motor imagery further, the multi-stimuli-cued unilateral lower limb motor imagery is studied in this paper. The visual-auditory evoked pathway is proposed and the sensory process is studied. To analyze the visual-auditory interactions, the kinesthetic motor imagery with the visual-auditory stimulus, visual stimulus and no stimulus are involved. The motor-related rhythm suppression is applied on quantitative evaluation. To explore the statistical sensory process, the causal relationships among the functional areas and the event-related potentials are investigated. The results have demonstrated the outstanding performances of the visual-auditory evoked motor imagery on the improvement of the mirror neuron system activation and the motor imagery ability. Besides, the abundant information interactions among functional areas and the positive impacts of the auditory stimulus in the motor and the visual areas have been revealed. The possibility that the sensory processes evoked by the visual-auditory interactions differ from the one elicited by kinesthetic motor imagery, has also been indicated. This study will promisingly offer an efficient way to motor rehabilitation, thus favorable for hemiparesis and partial paralysis patients.

## Introduction

Motor imagery (MI) is a type of mental simulation of motor behavior and however, without any actual execution (Qiu et al., 2017). The premotor area, the primary motor area, the somatosensory motor area, and the cerebellum have been reported to be activated in MI as motor execution (Taube et al., 2015). MI based brain computer interface (BCI) has been widely studied in motor rehabilitation (Xu et al., 2014) and physical disability assistance (Choi and Kang, 2014). For MI depends on the participant's imagination ability which is individual difference, the motor ability acquirement has been limited (Cirstea et al., 2003). Mirror neuron system (MNS) is a series of visuomotor neurons, and it is first discovered in F5 area of macaque (Cattaneo and Rizzolatti, 2009). MNS is considered as the physiology basics of the prediction of the action's effects (Knoblich and Flach, 2010). It also plays an important role in motor skills acquirement (Garrison et al., 2010). MI is mediated by the MNS (Babiloni et al., 2003; Eaves et al., 2014). MI ability is believed to represent the ability to arrange movement and to utilize internal forward model for the prediction of the motor outcome before the available sensorimotor feedback (Reynolds et al., 2015). MI ability has been identified to benefit from the MNS activation by researches on hand, mouth, and foot in human (Dickstein and Deutsch, 2007). MI is reported to improve motor performance by the promotion of MNS activation (Gatti et al., 2013) and to generate changes in structure and function of high-order motor cortical areas, (Slagter et al., 2011). Hence, it has been considered to be an effective way of the motor performance improvement. In order to improve the MNS involvement and MI ability, many researches concerning on stimulus evoked MI have been carried out. The effectiveness of the improvement on the MI performance by visual stimulus has been revealed (Hanakawa et al., 2003). An abundant guidance information provided by the auditory stimulus is demonstrated (Schreuder et al., 2010). A promising way of MI ability improvement has been reported by a video-cued unilateral lower limb MI (Boord et al., 2010). The MNS involvement has been improved by the object-oriented visual stimulus (Li et al., 2015). The MNS has been proved as a crucial role during the ecological stimuli (Murgia et al., 2015, 2016, 2018; Sors et al., 2015; Pizzera et al., 2017), and furthermore, the ecological visual and auditory stimuli can effectively affect complex movements (Kennel et al., 2014; Camponogara et al., 2016; Murgia et al., 2017). The rhythmic auditory stimulation is also indicated to facilitate gait rehabilitation (Thaut et al., 1993, 1996; Pau et al., 2016; Dalla Bella et al., 2017; Bailey et al., 2018). The possibility of achieving better performances on brain wave response and information transfer rate (ITR) by rich multi-sensory synergism is indicated (Moonjeong et al., 2012). Nevertheless, within our knowledge, the ability improvement approach and the sensory process of the multi-stimuli-cued unilateral lower limb MI are still not clear.

The mu frequency oscillation within the range of 8–12 Hz is relevant to the MNS activity (Pfurtscheller et al., 2008; Lapenta and Boggio, 2014). The beta frequency within the range of 13–30 Hz may be also related to the motor-related neuron activity (Li et al., 2015). During MI, electroencephalogram (EEG) desynchronization resulting from thalamocortical stimulus is a reliable correlate of the activated cortex, while EEG synchronization is a correlate of the deactivated cortex (Wriessnegger et al., 2013). The event-related desynchronization (ERD) and synchronization (ERS) on mu and beta frequencies are the indexes of the MNS involvement and the MI ability (Perry et al., 2011).

Event-related potential (ERP) is the neurophysiological activity that responds to the sensory stimulation in the background EEG. ERP can be divided into the endogenous and exogenous components. The endogenous component provides a sensitive measurement to assess information processing. The most studied endogenous component P300 is elicited by infrequent novel stimulus (P3a) and/or infrequent target stimulus (P3b). It reflects high-order information processing associated with the contextual evaluation of attended stimuli. The latency of the P300 indicates the time taken for the activation (Wang et al., 2003). The exogenous component is related to the attention and the sensory processing. One of the most studied exogenous components is N100. This is activated by several intra-cranial generators and is regarded as the reflection of the general and nonspecific cerebral excitability (Cortoos et al., 2014). In the neurophysiological research, the neural mechanism underlying the cognitive process can be reflected by the precise timing of ERP.

To improve the activation of MNS and the MI ability further, the multi-stimuli-cued unilateral lower limb MI is studied in this paper. In view of the positive performance with the visual stimulus, the effects of visual-auditory interactions on lower limb MI and the sensory process are investigated. The suppressions of mu and beta EEG oscillations and the ERP are applied for quantitative evaluation and analysis. This work devotes to explore the underlying neural mechanism of multi-stimuli-cued lower limb MI, and hopefully to provide an efficient path for motor rehabilitation especially lower limb rehabilitation, thus favorable for hemiparesis and partial paralysis patients.

## Materials and methods

### Participants

In this study, 10 participants composed of 8 males and 2 females with the mean age of 22.4 ± 1.43 years old are involved. They are able-bodied and free from medication and any disorders of or injuries to the central nervous system. The study is approved by the ethics review board of Northeastern University and is conducted according to the declaration of Helsinki. Studies are implemented after signing written consent forms by participants.

### Recordings

**Figure 1:**
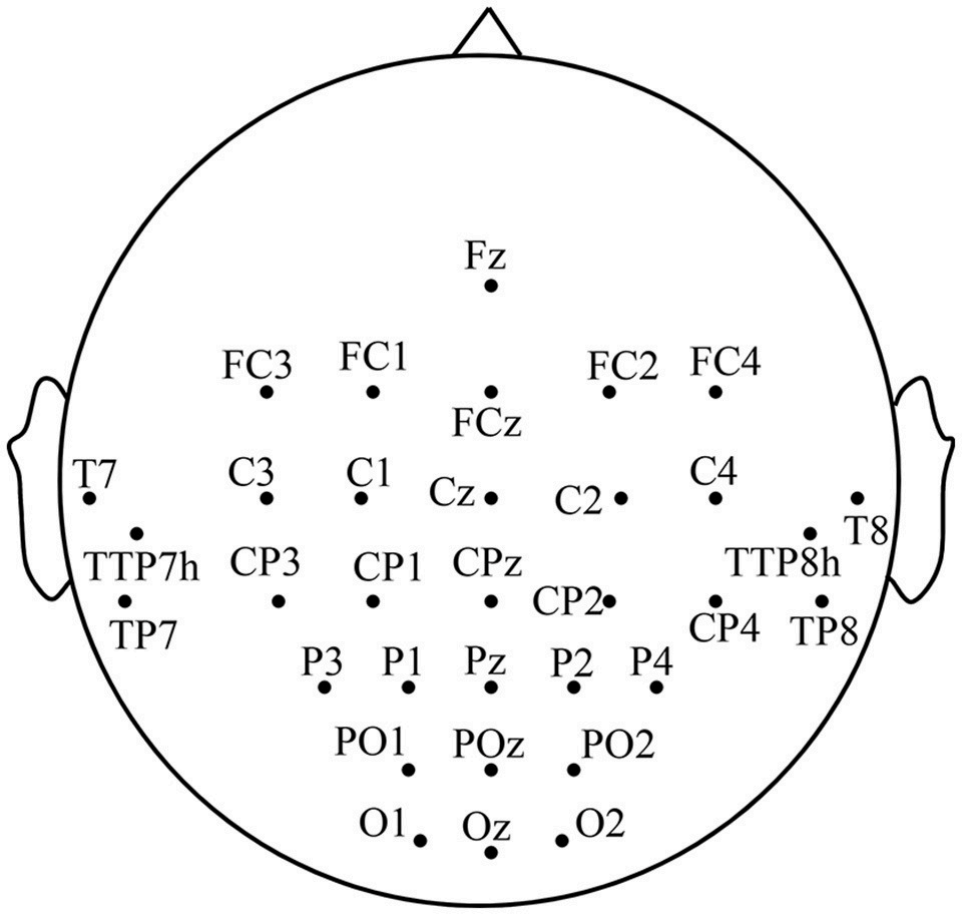
The distribution of the electrodes.

EEG signals are recorded with 32 Ag-AgCl active electrodes including the motor area, the visual area and the auditory area, with the g.HIamp (g.tec Inc., Austria) system according to the 10-5 electrode location system (Jurcak et al., 2007). The motor area consists of the premotor and supplementary motor cortex (A6), the primary motor cortex (A4), and the primary somatosensory and somatosensory association cortex (A1-2-3-5), etc. The visual area is composed of the primary visual cortex (A17), the secondary visual cortex (A18) and the associative visual cortex (A19). The distribution of electrodes is illustrated in Figure 1. EEG signals are referenced to a unilateral earlobe and grounded at frontal position (Fpz) with a sampling rate of 1,200 Hz. To suppress artifacts and power line interference, online band-pass filter between 2 and 100 Hz and notch filter between 48 and 52 Hz are applied on the recorded EEG. All impedances of active electrodes are kept below 30 kΩ during experiments. To avoid the influences of electromyographic (EMG), the differential voltages between EMG electrode pairs on rectus femoris and biceps femoris of each leg are also recorded using the g.HIamp system with a sampling rate of 1,200 Hz. The EEG trials with any actual leg movement are discarded from further analysis to avoid the EMG disturbance.

### Experimental procedures

**Figure 2:**
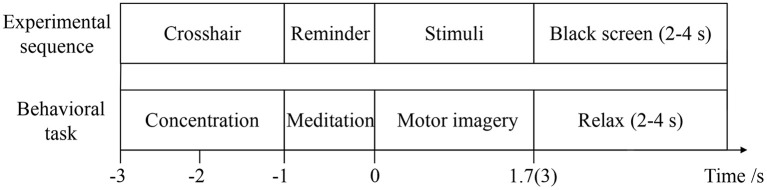
The details of the trial.

To evaluate the effect of visual-auditory interactions on lower limb MI, three kinaesthetic MI tasks, “visual-auditory context,” “visual context,” and “imagery context,” are conducted. The visual stimulus that is applied on the “visual-auditory context” and the “visual context,” presents the extension and restoration movements of the unilateral leg using the 1.7 s color video frames. The binaurally auditory stimulus, extending leg and restoring it, is introduced using the 1.7 s recordings of native language and it is applied to the “visual-auditory context.” During the “visual-auditory context,” participants are instructed to imagine the unilateral leg extension and restoration movements with the paralleled visual and auditory stimuli of the same direction. During the “visual context,” participants are instructed to imagine the same movements accompanied by visual stimulus. During the “imagery context,” participants are instructed to imagine the same movements without any stimulus. The experiments are conducted in a dark and electrically shielded room. Participants are seated in an armchair comfortably with the distance of 95 centimeters between nose and computer screen approximately. There is a half an hour training session for each participant to be familiar with the trial design by motor execution before experiments. The three tasks are presented in pseudorandom for participants. The trials of the experiments are shown in Figure 2. The trials start with a crosshair that lasts for 2 s at the center of the screen. Participants are required to focus on the crosshair to reduce ocular movement. Subsequently, the arrowhead randomly pointing to left or right at the center of the screen lasts for 1 s as a reminder. When the arrowhead disappears, the stimulus or blank appears. Meanwhile, participants are instructed to perform the kinaesthetic MI. The imagery processes of the “visual-auditory context” and “visual context” last for 1.7 s. With regard to the third task, the imagery process lasts for 3 s in view of the initiative MI. There is a random break of 2–4 s at the end of each trial and a 1-min break after every ten trials for rest. Each run is comprised of five trials for the left and five trials for the right leg. During every task, 75 trials for each leg of the participants are implemented. Presentation of the visual, auditory, blank and their reversals are controlled by the psychophysics toolbox 3.0 (Brainard, 1997).

### Analysis

To reduce the ocular artifacts, the EMD-regression algorithm (Li et al., 2013) is employed. All trials are visually inspected based on EMG during MI process, and nearly 9% contaminated trials are discarded from further analysis. To reduce the influences of the volume conduction and the reference electrode selection (Li et al., 2015), as well as to improve the spatial resolution and the signal-to-noise ratio of EEG, the surface Laplacian is applied (Boord et al., 2010). Subsequently, all trials are extracted from data flow. To observe the brain activity, the average suppression index (ASI) illustrated by Equation (1), is applied. It is the average power ratio of the imagery process and the baseline (Pfurtscheller and da Silva, 1999). In this study, the EEG signals between −2.5 and −1.5 s of each trial are used as the baseline. As there is no relative information of kinesthetic MI during the crosshair and reminder process, only the imagery process of the three tasks is analyzed in this study.

(1) $$\begin{array}{l} ASI(f)=\frac{1}{p+1}\sum_{t_{a}=t_{oa}}^{t_{oa}+p}(\frac{1}{n}\sum_{i=1}^{n}I^{2}(f,\;t_{a})\\ \;-\frac{1}{k+1}\sum_{t_{b}=t_{o}}^{t_{o}+k}\frac{1}{n}\sum_{i=1}^{n}R^{2}(f,\;t_{b}))/\frac{1}{k+1}\sum_{t_{b}=t_{o}}^{t_{o}+k}\frac{1}{n}\sum_{i=1}^{n}R^{2}(f,\;t_{b}) \end{array}$$

where, *I*(*f*, *t*) and *R*(*f*, *t*) denote the imagery process and baseline on the concerned frequencies *f* ; *n* is the trial number; *k and p* are in connection with the point number of baseline and imagery process.

The relationships of the visual, auditory and motor areas during different tasks are studied using the Granger causality analysis to explore the neural meditation mechanism and the sensory process evoked by the visual-auditory interactions. The Granger causality analysis is a statistical measurement based on the time sequence forecast. If the past information from one time sequence is benefit to a better prediction accuracy of another sequence, the first sequence has a causal influence on the second one. Due to the mutual interactions elicited by the volume conduction and the multi-electrodes, the multiple vector autoregressive (MVAR) model of the Granger causal analysis (Seth, 2010) is applied in this study. The ratio of the Akaike information criterion to the Bayesian information criterion is used to calculate the order of the MVAR model.

### Statistics

To evaluate the differences of the MI abilities and the MNS activation during the three tasks, the analysis of variance (ANOVA) is applied to analyze the ASI of the imagery process. ANOVA is employed on mu and beta frequency oscillations to evaluate the differences of the three tasks in each functional area and to analyze the functional differences. The factors are within-subjects factors, “condition” (“visual-auditory context” vs. “visual context” vs. “imagery context”), “rhythm” (mu and beta) and “area” (A6, A4, A1-2-3-5, A17, A18, A19, and auditory area). To study the differences of the three tasks in the contralateral hemisphere and in the ipsilateral hemisphere, the mu and beta ASIs are analyzed by ANOVA. The factors are “condition” (“visual-auditory context” vs. “visual context” vs. “imagery context”), “area” (motor area, visual area and auditory area), “rhythm” (mu and beta) and “hemisphere” (contralateral vs. ipsilateral). To evaluate MI abilities by the differences between the contralateral and ipsilateral hemispheres, ANOVA is applied on the mu and beta ASIs. The factors are “condition” (“visual-auditory context” vs. “visual context” vs. “imagery context”), “area” (motor area, visual area and auditory area), “hemisphere” (contralateral vs. ipsilateral) and “rhythm” (mu and beta). Moreover, the ERP differences of the three tasks are also analyzed by ANOVA. The peaks of ERPs are adopted. The factors are “condition” (“visual-auditory context” vs. “visual context” vs. “imagery context”), “ERP” (P2, N1, N2, and P3) and “electrode” (Fz, Cz, Oz, T7, and T8). All the analysis and calculation are performed using MATLAB.

## Results

**Figure 3:**
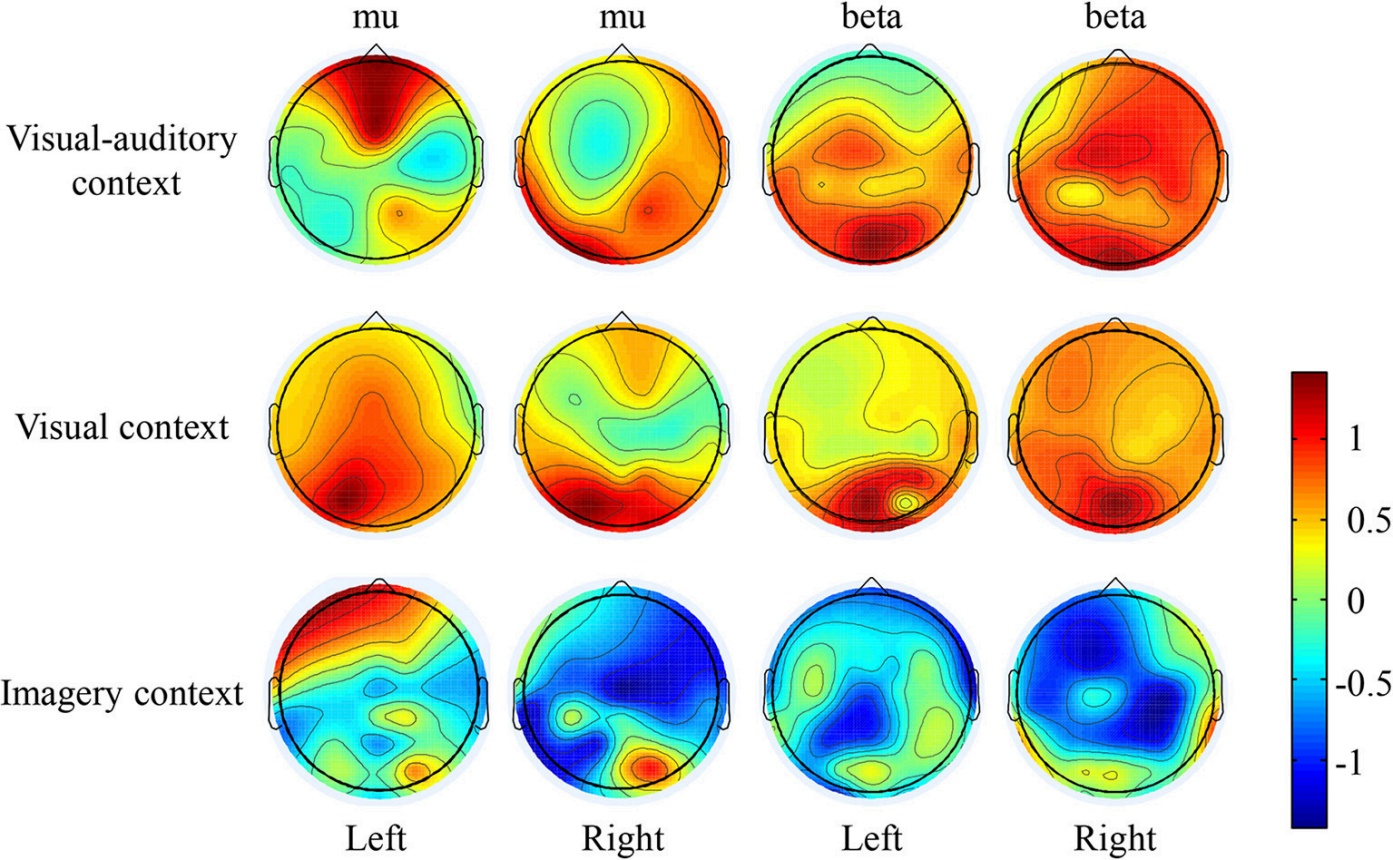
The ERD/ERS topographical view of the mu and beta frequencies.

**Figure 4:**
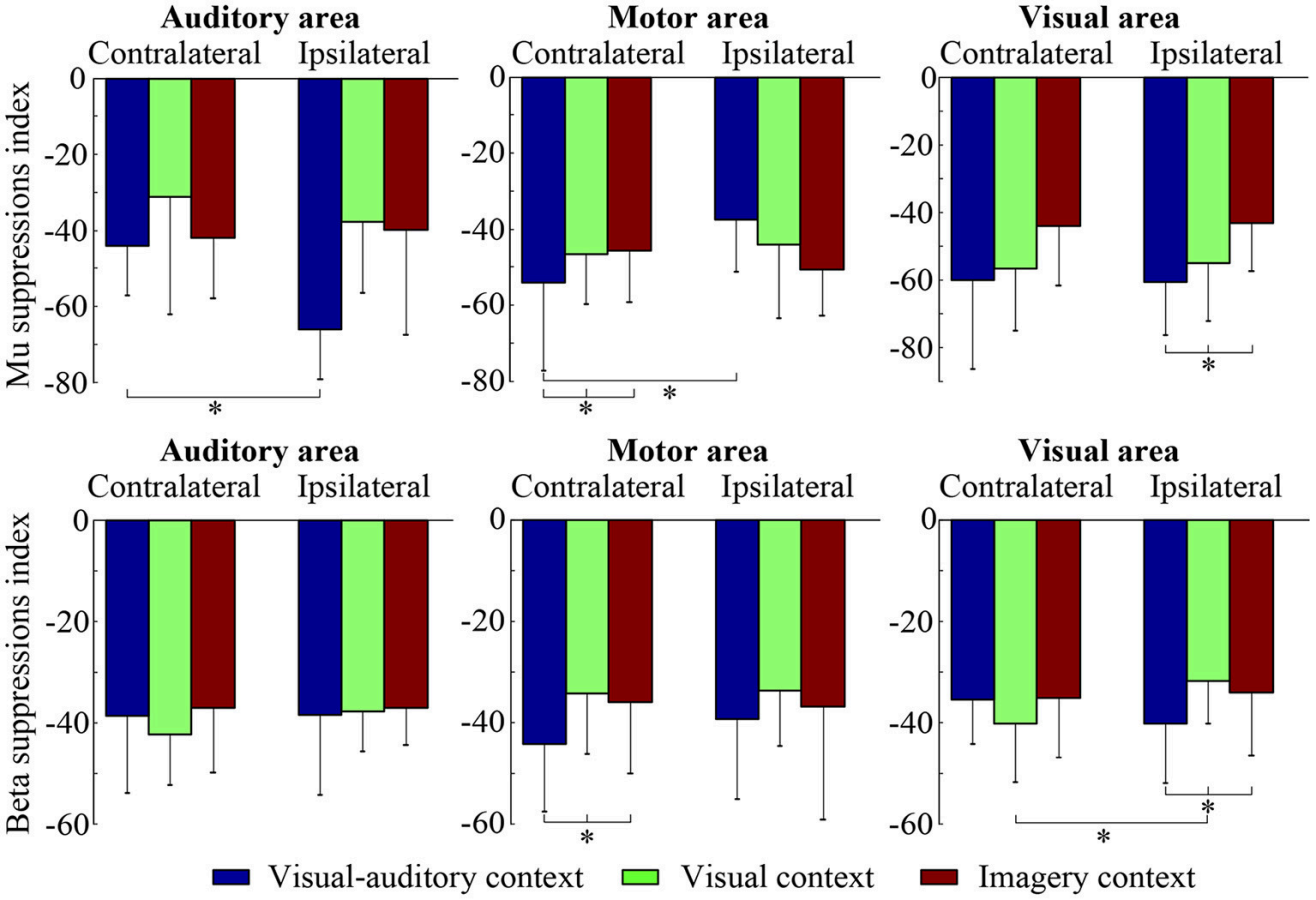
The statistical results of the three tasks on the contralateral and ipsilateral hemispheres. The * indicates significant difference (*p* < 0.05).

The suppressions of mu and beta frequencies are applied to analyze the cortical excitement. The topographical views of the average ERD/ERS on the mu and beta frequencies during the three tasks are illustrated in Figure 3. Under the “visual-auditory context” and the “visual context,” the unilateral leg MI provides the mu and beta suppressions in the contralateral hemisphere. In the “imagery context,” the mu suppression can be found at the central area. The statistical results of the three tasks in the functional areas present significant mu differences in the A6 area [*F*_(2, 98)_ = 14.62 *P* < 0.05], the primary motor area [*F*_(2, 98)_ = 8.72 *P* < 0.01], the primary visual cortex [*F*_(2, 58)_ = 14.47 *P* < 0.01], and the auditory area [*F*_(2, 118)_ = 5.84 *P* < 0.01]. Besides, there are significant beta differences of the three tasks in the A6 area [*F*_(2, 98)_ = 29.18 *P* < 0.01] and the primary motor area [*F*_(2, 98)_ = 26.05 *P* < 0.01]. ANOVA results of the three tasks in the contralateral hemisphere and in the ipsilateral hemisphere indicate that both of the mu and beta suppressions are significantly different in the contralateral motor area {mu: [*F*_(2, 238)_ = 18.72 *P* < 0.001], beta: [*F*_(2, 238)_ = 6.56 *P* < 0.01]} and in the ipsilateral visual area {mu: [*F*_(2, 158)_ = 3.13 *P* < 0.05], beta: [*F*_(2, 158)_ = 4.02 *P* < 0.05]}. The statistical results between the contralateral and ipsilateral hemispheres demonstrate the significant mu differences between the contralateral and ipsilateral hemispheres in the motor area [*F*_(1, 119)_ = 5.67 *P* < 0.05] and the auditory area [*F*_(1, 59)_ = 8.23 *P* < 0.01] under the “visual-auditory context.” The “visual context” presents a significant beta difference in the visual area [*F*_(1, 79)_ = 6.92 *P* < 0.05]. Other comparisons by ANOVA which are not listed, are not significant difference (*P* > 0.05). The statistical results are shown in Figure 4.

**Figure 5:**
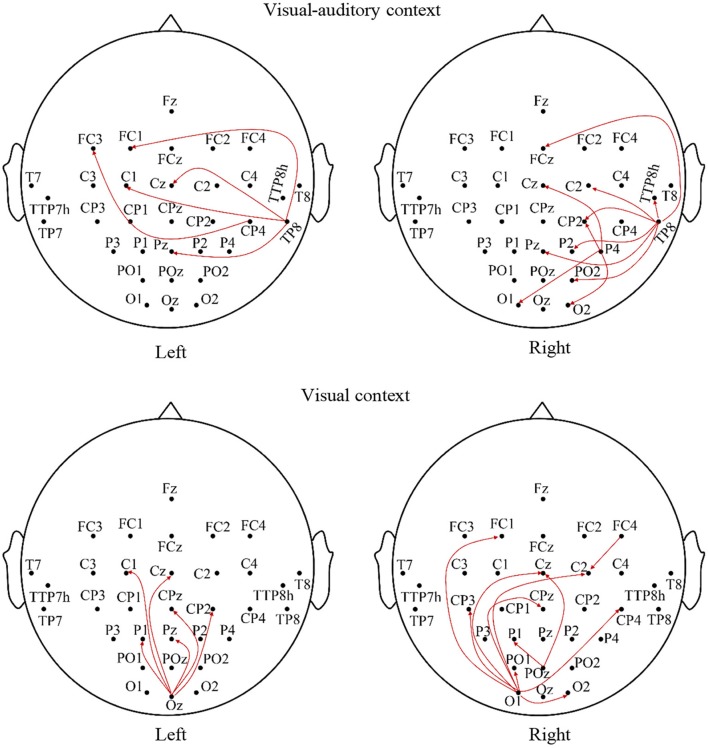
The average Granger causality results of the imagery process of the participants during the visual-auditory context and visual context.

The relationships of the visual, auditory and motor areas under the “visual-auditory context” and the “visual context” are studied by the Granger causality analysis to explore the underlying neural mechanism evoked by stimulus. The average analysis results of the imagery process of the participants are illustrated in Figure 5. The connectivity between electrodes presents a significant connection (*P* < 0.01). The analysis results demonstrate the causal influences from the auditory area of the right hemisphere to the visual and motor areas, and from the visual area to the motor area under the “visual-auditory context.” Besides, the causal connectivity from the visual area to the motor area under the “visual context” is also revealed.

**Figure 6:**
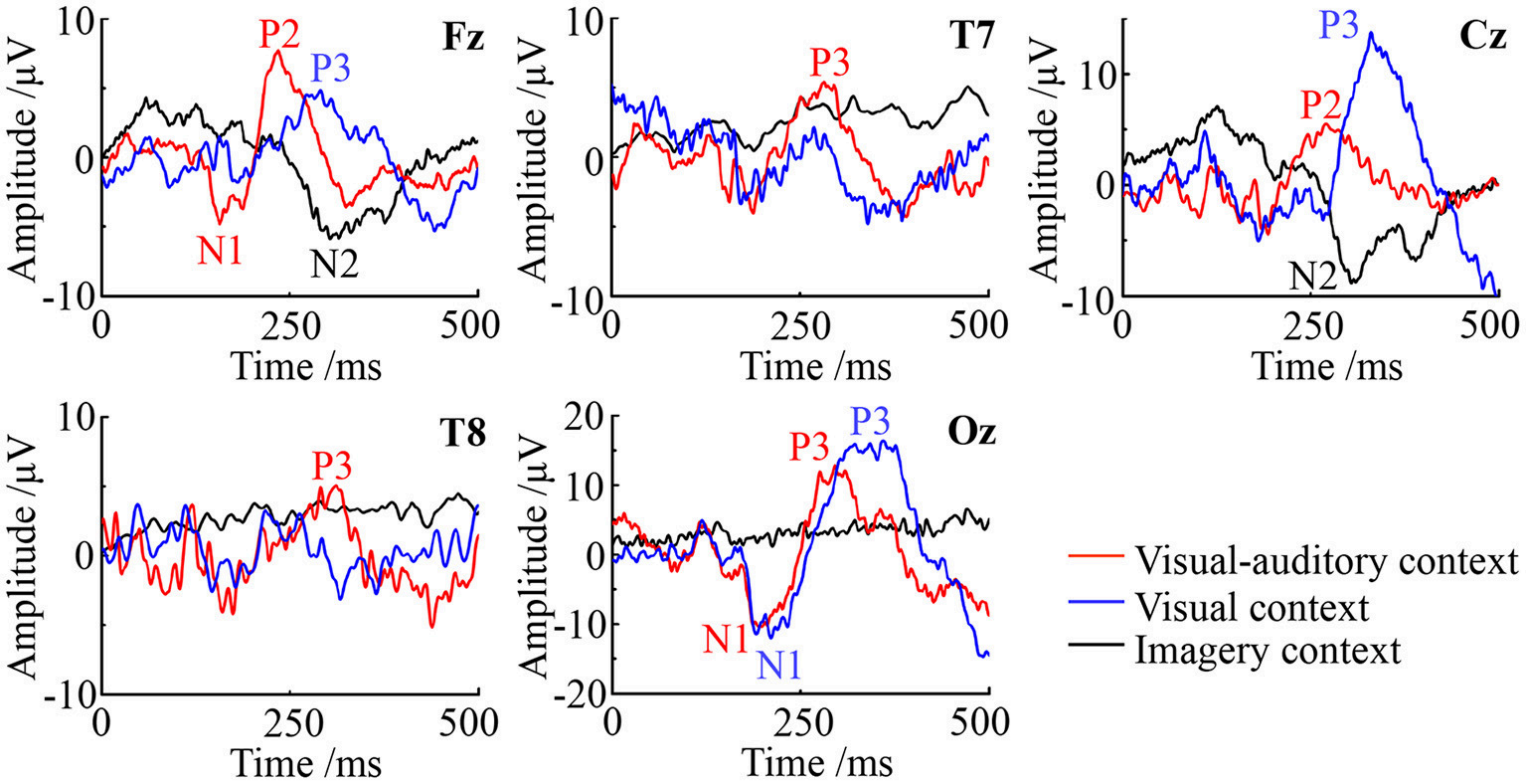
The ERP waveforms of the visual-auditory, visual and imagery contexts.

ERPs on Fz (frontal), Cz (central), Oz (posterior), T7 (left) and T8 (right) are studied to evaluate the potential neural mediation during the imagery process. The average ERP waveforms of the three tasks on these electrodes are illustrated in Figure 6. In the figure, the red line, blue line and black line represent the “visual-auditory context,” “visual context,” and “imagery context” respectively. In the “visual-auditory context,” there are N100 (N1) on Fz and Oz, P200 (P2) on Fz and Cz, P300 (P3) on Oz, T7 and T8. In the “visual context,” P3 and N1 are found on Oz. Meanwhile, P3 can be also discovered on Fz and Cz. In the “imagery context,” N200 (N2) is found on Cz and Fz. The statistical results of the three tasks demonstrate the significant differences of the three tasks on P2 [*F*_(2, 98)_ = 119.97 *P* < 0.05], N1 [*F*_(2, 98)_ = 22.51 *P* < 0.05], P3 [*F*_(2, 98)_ = 78.3 *P* < 0.05], and N2 [*F*_(2, 98)_ = 24.49 *P* < 0.05]. There is a significant difference among the four kinds of ERPs [*F*_(3, 447)_ = 521.42 *P* < 0.05]. In addition, there is a significant interaction of “condition” × “ERP” [*F*_(6, 249)_ = 20.76 *P* < 0.05] that indicates the significant ERP difference among conditions.

## Discussion

The mu rhythms which are originated at parietal lobe, are attenuated during attending motor behavior (Gastaut, 1952), such as motor imagery (Pfurtscheller et al., 2006). Many studies have suggested that the desynchronization and attenuation in mu rhythm activity reflect MNS modulation (Muthukumaraswamy et al., 2004; Oberman et al., 2005). Therefore, the mu rhythm has been treated as a physiological indicator of MNS (Honaga et al., 2010). The suppressions of beta rhythms which originate from the precentral cortex, have also been regarded as indicators of MNS (Honaga et al., 2010) and motor behavior (Bai et al., 2008). Based on the above, the suppressions of mu and beta rhythms are related to the mirror neurons activation and the MI ability. In this study, the mu and beta suppressions have been discovered in the MI process under the “visual-auditory context” and the “visual context.” There are significantly different mu and beta suppressions among the three tasks in the A6 and the primary motor area. These suggest the differences of the motor neuron mediation among these tasks, and a greater MNS activation evoked by stimulus. Both of the mu and beta contralateral suppressions have presented significant differences in the motor area among the three tasks. In addition, the mu rhythm has exhibited a significant difference between the ipsilateral and the contralateral motor areas under the “visual-auditory context.” The above results reveal the greater motor-related rhythm suppression under the “visual-auditory context” than under other tasks. Namely, the visual-auditory interactions can promote the MNS activation and the MI ability. The MNS plays a crucial role during MI evoked by the visual-auditory interactions. The MNS theory provides a path to study motor behavior. This mirror-like system has been convinced that it contributes to the social behavior by many researches (Wicker et al., 2003; Leslie et al., 2004). MNS is suggested to be involved in complex functions except for motor interpretation. It is constrained by motor mode and is differently encoded (Cattaneo and Rizzolatti, 2009). Hence, the results of this study indicate that the visual-auditory interactions may activate more perceive activities than other tasks by the promotion of MNS activation.

The activation of the auditory cortex is closely related with memory-scanning task (Krause et al., 1995). In the study of mu and beta frequency oscillations, significant mu difference among the three tasks in the auditory cortex has been revealed. Only under the “visual-auditory context,” a significant mu difference between the ipsilateral and the contralateral auditory cortices has been verified. These results reveal that the mu rhythm fluctuation may have a relationship with the activity of the auditory cortex, and a greater ipsilateral-auditory mu suppression has been presented by the binaural stimulation. During MI process evoked by the visual-auditory interactions, the activation of the auditory cortex that may be involved in the memory recall activity of the brain is asymmetrical. Besides, the significant differences of the mu suppression in the primary visual cortex and of the mu and beta suppressions in the ipsilateral visual area have been indicated among the three tasks. That is, the activation of the visual area varies with the tasks. Both of the mu (alpha) and beta rhythms may be affected by the visual stimulus. Among the three tasks, a significant beta difference between the ipsilateral and the contralateral visual areas has been discovered only under the “visual context.” Namely, the introduction of the auditory cue may suppress the beta difference between the ipsilateral and the contralateral visual areas under the “visual-auditory context.” As a result, the auditory area activation may have an effect in the visual area.

With the aim to explore the relationship of the motor, auditory and visual areas during the MI process evoked by stimulus, the Granger causality analysis has been employed. The results concerning the “visual-auditory context” have indicated that both of the motor and visual areas are affected by the auditory area of the right hemisphere. Besides, the motor area is also affected by the visual area. Under the “visual context,” there is a causal effect from the visual area to the motor area. The study about visual-auditory interactions (Molholm et al., 2002) indicates the possibility of the impact of the auditory stimulus in the auditory and visual areas and of the impact from the primary auditory or the auditory cortex to the visual cortex during the button-press response task under the auditory and visual instructions. The relative anatomy research indicates that some axons of the visual cortex pass by the thalamus, and end in mesencephalon (Benevento, 1975). The mesencephalon is relevant to the reflection of the visual and auditory stimuli. This may be the anatomical basis of the causal connection from the auditory cortex to the visual cortex under the “visual-auditory context.” The auditory area in the right hemisphere plays a predominant role in the attention control (Heilman and Van Den Abell, 1980) and the listening task without any specific strategies (Tervaniemi and Hugdahl, 2003). As a result, it has dominated the causal connections from the auditory to the visual and the motor areas under the “visual-auditory context.” Based on the outstanding MI ability, the causal connections indicate a positive effect of the auditory cortex in the motor and visual cortices under “visual-auditory context.” This results demonstrate that the auditory stimulus may activate the similar cognitive process by memory recall with the one by visual stimulus and kinesthetic MI, as the auditory cortex activation is closely related with memory recall (Krause et al., 1995). The dorsal pathway of the visual cortex is not a strict serial hierarchy. While, in general, A17 accepts the nervous discharge from the lateral geniculate nucleus. Then the projections extend to A18 and A19, and finally reflected in the somatosensory area by A18 and A19, etc. (Van den Stock et al., 2011). Accordingly, the significant causal connection from the visual area to the motor area under the “visual-auditory context” and the “visual context” may indicate the information transmission process of the dorsal pathway evoked by the visual stimulus.

ERPs with a high temporal resolution offer a sensitive path to monitor brain electrical activity and to observe cognitive process (Delle-Vigne et al., 2014). In this study, significantly different EPRs are presented among the three tasks. Furthermore, there is a significant interaction of “condition” × “ERP.” Namely, the brain activity and the cognitive process vary with tasks. Under the “visual-auditory context” and the “visual context,” N100 and P300 emerge on Oz, while these ERPs can not be observed under the “imagery context.” N1 has been proved as a type of the visual evoked potentials, which can be elicited by visual stimulus. It is significantly affected by the early phase of perception and attention processing (Bar-Haim et al., 2005). As a result, the ERPs of the N100 and P300 above may be the response of visual stimulus and the reflection of the visual area's activity. Besides, N1 is also thought to be evoked by the auditory stimulus (Annic et al., 2014). This auditory N1 is a measurement of the initial registration, the affiliation selection and the process of the auditory stimulus (Woldorff et al., 1987). Therefore, in view of the causal influence from the auditory area to the motor and visual areas, N100 on Fz and Oz may be also the reflection of cognitive process evoked by the visual-auditory interactions under the “visual-auditory context.” The P200 elicited by auditory stimulus presents over the vertex (Cz) prominently, with a typical peak latency of 150–250 ms approximately (Ferreira-Santos et al., 2012). It reflects the later stage of the stimulus processing, regarded as an index of some aspects of the stimulus classification process (Annic et al., 2014). The P200 only observed under the “visual-auditory context” may be the response of the recognition process evoked by auditory stimulus. During the auditory stimulus processing, the frontal lobe and the parietal lobe may be involved. N2 as a type of cognitive potential can be only observed under the “imagery context.” The sensory process of the “imagery context” are different with the one of the other two tasks. The auditory and visual stimuli may convert the cognitive process of kinesthetic MI.

## Conclusion

With the aim to explore the effect of the visual-auditory interactions on lower limb MI and the sensory process, three kinds of kinesthetic MI have been involved in this study. The study results have demonstrated the noteworthy performances of the visual-auditory evoked MI on the improvement of the mirror neurons activation and the MI ability. The visual-auditory evoked MI has presented the abundant information interactions among the functional areas and the positive impacts of the auditory stimulus on the motor and visual areas. Besides, the study results also reveal that the cognitive process of kinesthetic MI may be converted by the visual-auditory and visual stimuli.

The hemiparesis and partial paralysis are the common sequelae after stroke, affecting the daily life quality of patients directly. To recover the patients' somatic and sensory motor abilities, MI assisted therapy is a promising path of motor rehabilitations. This study about the visual-auditory interactions on lower limb MI will be favorable for motor learning and rehabilitation. Other imaging technology of brain will be explored to study the effect of visual-auditory interactions in further work, in order to overcome the low spatial resolution of EEG.

## Author contributions

The data was analyzed by ZY and LL, the paper written by ZY, the materials and analysis tools supplied by ZY and LL, the language corrected by JS and HL.

### Conflict of interest statement

The authors declare that the research was conducted in the absence of any commercial or financial relationships that could be construed as a potential conflict of interest.
